# Visualizing Research on Industrial Clusters and Global Value Chains: A Bibliometric Analysis

**DOI:** 10.3389/fpsyg.2020.01754

**Published:** 2020-07-22

**Authors:** Thais González-Torres, José-Luis Rodríguez-Sánchez, Antonio Montero-Navarro, Rocío Gallego-Losada

**Affiliations:** ^1^Department of Business Economics, Applied Economics II and Fundamentals of Economic Analysis, Rey Juan Carlos University, Madrid, Spain; ^2^Department of Financial Economy and Accountancy, Rey Juan Carlos University, Madrid, Spain

**Keywords:** global value chain, industrial cluster, bibliometric study, co-citation analysis, co-word analysis, Sci-mat, VOS-viewer

## Abstract

In the current digital era, the borders amongst firms are getting blurred when it comes to value creation. Therefore, the traditional configuration of the value chain is frequently replaced by other ones which include the collaborative participation of different agents. Within this context, global value chains, where the value activities are located in different countries, and industrial clusters, which combine competition and cooperation, are attracting a growing attention of both business leaders and scholars in the recent years. Through a bibliometric analysis, this paper disentangles the intellectual and conceptual structure of the research topic of industrial clusters and global value chains. Results show the multidisciplinary character of the topic, including papers published in different areas, such as business, regional studies and world development, as well as its close link with aspects like innovation, regional development, governance or organization. Finally, this study remarks the research lines that could attract more attention in the immediate future.

## Introduction

From the seminal work of Prahalad and Ramaswamy (2000) and the proposal of a service-dominant logic presented by Vargo and Lusch (2004), many authors have worked in the field of value cocreation, giving birth to an abundant and varied literature (Ranjan and Read, 2016) which stems from overcoming a linear vision of value chains and value creation. From a value cocreation perspective, as stated by Ballantyne (2004), the traditional image of a supplier producing goods and services to be offered to the customers is replaced by an interactive process of learning together, where the participants integrate their resources and competencies to increase the creation of value in a service system (Vargo et al., 2008).

Different works have emphasized the relevance of the competitive and cooperative environment when it comes to empower value cocreation. Ngongoni and Grobbelaar (2017) studied how the participation of external agents spurred value cocreation in an entrepreneurial ecosystem. Rupo et al. (2018) studied an Italian maritime cluster from different perspectives, including value cocreation. Finally, Crick et al. (2020) analyzed the importance of competitor orientation in a wine cluster to foster enhanced value cocreation activities. Thus, from a service-dominant logic, industrial clusters and global value chains can be good examples of the rupture of the traditional linear supplier–customer relationship.

Industrial clusters enable companies to specialize in their core business by providing them with a wider network of specialized suppliers and providers, skilled workers and business associations (Giuliani et al., 2005), enabling the integration of resources and competencies. A cluster or industrial district requires geographic proximity and sectorial concentration of organizations in order to seek complementarities that allow them to generate competitive advantages (Porter, 1990, 2000). However, the production of products and services is increasingly seen as a process that takes place where the necessary resources are available in a competitive manner, both in terms of cost and quality. In this context, the approach of the global value chain arises as a tool enabling the consideration of the operations developing outside the cluster and the key role of relations with the most relevant external agents (Gereffi, 1999; Giuliani et al., 2005).

Industrial clusters and global value chains have attracted the interest of both business leaders and scholars in different disciplines. Academic research has grown rapidly because these strategies are increasingly considered as potential ways to enhance firm competitiveness in international markets (Giuliani et al., 2005). Considering the expansion of the field in recent years, the need for a systematic review of the existing literature arises.

In order to fill this gap, the main goal of this article is to provide a review of research on industrial clusters and global value chains. Accordingly, the specific objectives are the following: (1) to assess the productivity, impact and relative influence of the research topic; (2) to present the intellectual structure; (3) to identify the thematic organization and (4) to identify the conceptual structure. According to these goals and following recent bibliometric reviews, we can also settle the following research questions (González-Torres et al., 2020):

– RQ1: What is the historical evolution of the literature of industrial clusters and global value chains?
– RQ2: Which are the most productive journals addressing industrial clusters and global value chains?
– RQ3: Who are the most productive authors addressing industrial clusters and global value chains?
– RQ4: Which are the most prominent documents in the field of industrial clusters and global value chains?
– RQ5: Which are the main documents that have influenced the intellectual structure of the topic industrial clusters and global value chains?
– RQ6: Which are the main journals around which the research topic of industrial clusters and global value chains is organized?
– RQ7: What are the top subjects and topics in the field of industrial clusters and global value chains?

In order to answer these questions, a qualitative and quantitative analysis was performed using a bibliometric methodological approach, following other relevant works (Gomezelj, 2016; Marasco et al., 2018). Bibliometric analysis helps scholars draw useful conclusions about a specific research field by analyzing citations, co-citations and word frequency, among others. The appropriate bibliometric method has been chosen to encompass a broad picture of the literature (see Table 1).

**TABLE 1:** Bibliometric methodology.

Objective	Research questions	Bibliometric method	Analysis	Software
(1) To assess academic impact and relative influence	RQ1: Historical evolution literature	Evaluative techniques: Productivity Assessment	Historical evolution of documents	Sci-Mat
	RQ2: Most productive journals		Distribution of documents by journal	
	RQ3: Most productive authors		Distribution of documents by author	
	RQ4: Most prominent documents	Evaluative techniques: Impact metrics	Citation analysis	
(2) To determine intellectual structure	RQ5: Main documents influencing intellectual structure	Relational techniques: co-citation	Co-citation analysis: documents	VOS -Viewer
			Co-citation analysis: authors	
(3) To identify thematic organization	RQ6: Main journals around which the research topic of is organized		Co-citation analysis: journals	
(4) To identify conceptual structure	RQ7: Patterns and hot topics	Relational techniques: co-occurrence	Co-word analysis	

Firstly, we have used the software Sci-mat (Cobo et al., 2012). This software allows us to apply evaluative techniques of academic productivity and impact metrics. Thus, productivity is measured according to the historical evolution of published articles, the distribution of articles by journal or book and individual author. Academic impact or influence is analyzed according to the total number of citations of the most cited articles.

In addition to evaluative techniques, relational techniques are applied. Following a bibliometric mapping approach throw the VOS-Viewer software (Van Eck and Waltman, 2010) it is possible to detect the structure of the research topic. These results are presented in the form of visual networks through co-citation analysis —articles and journals— and co-words.

The main contribution of this article is to provide a synthesis of the body of research on industrial clusters and global value chains. Accordingly, we address the academic impact of the topic through diverse evaluative techniques of academic productivity and impact metrics. Finally, by using different relational techniques, we offer insights about the intellectual structure, thematic organization and conceptual structure of the subject. The relevance of this article lies in the importance of the research topic, which offers diverse challenging issues and research opportunities for many academic disciplines, gathering the interest of researchers mainly concerned about business management, development studies, and corporate social responsibility.

The organization of the article is the following: section “Theoretical Framework” provides a theoretical overview of the topic. In section “Materials and Method,” we explain the data collection and bibliometric methods used. Next section addresses the results of the evaluative and relational techniques. Finally, in the fifth and sixth sections, the main findings are presented, in the form of discussion, conclusions, limitations, and future research lines.

## Theoretical Framework

According to Morosini (2004, p. 307), an industrial cluster is a “*socioeconomic entity characterized by a social community of people and a population of economic agents localized in close proximity in a specific geographic region. Within an industrial cluster, a significant part of both the social community and the economic agents work together in economically linked activities, sharing and nurturing a common stock of product, technology and organizational knowledge in order to generate superior products and services in the marketplace.*” Being part of a cluster offers a wide range of potential benefits for businesses (Rodríguez-Sánchez, 2020). The main advantages are related to efficiency thanks to the division and specialization of the workforce, as well as the availability of qualified and trained workers. Moreover, from the perspective of external stakeholders, industry clusters give access to a broader range of providers and suppliers, to agents selling in national and international markets, and encourages the creation of business associations (Giuliani et al., 2005).

The capability of clustered firms to grow and be efficient has attracted great interest among scholars. However, the current globalization of markets and the widespread use of information and communication technologies (ICT) have transformed production process, distribution and sales systems, financial markets and even human talent retention (Schmitz and Nadvi, 1999; Kaplinsky and Gereffi, 2001; Rodríguez-Sánchez et al., 2020). The production of products and services is increasingly seen as a process that takes place where the necessary resources and capabilities are available in a competitive manner, both in terms of cost and quality (OECD, 2020). Accordingly, special focus should be given to external relationships, since the growing importance of export-oriented industrialization has made necessary for firms to integrate into a global economy (Gereffi and Kaplinsky, 2001).

In this context, the approach of the global value chain arises as a tool enabling the consideration of the operations developing outside the cluster and the key role of relations with the most relevant external agents (Gereffi, 1999; Giuliani et al., 2005; Lensen et al., 2012). The notion of value chain proposed by Porter (1990) helps to understand cost behavior and sources of differentiation and by disaggregating the company into its strategic activities. In addition, Porter (1990) highlights that the firm’s value chain is integrated into a broader flow of activities called the value system. While traditional approaches are limited to manufacturing, the value chain perspective broadens the focus to complementary activities in the production and provision of value-adding products and services, including distribution and marketing (Wood, 2001; Giuliani et al., 2005). Accordingly, it could be said that the vision of the value chain helps researchers to understand the characteristics and quality of the relationships between value chain agents (Humphrey and Schmitz, 2002).

Stemming from the basic notion of value chain and taking into account that firms are increasingly locating the different phases of the production process in different territories for competitive reasons, the concept of global value chain helps to understand how global production and distribution systems are organized (Humphrey and Schmitz, 2002). In this regard, for many industries, access to international markets means entering networks of international and diverse partners in areas such as design, production, and marketing (Gereffi and Kaplinsky, 2001).

From a service-dominant logic, as Ranjan and Read (2016), the final value is not necessarily created in a co-production activity, but also through the use of the product (value in use). As Alves et al. (2016) state, value co-creation takes place when the resources of one service system integrate with those belonging to another one. This is a typical situation in both clusters and global value chains, where value creation and knowledge acquisition are empowered by the interaction of the different agents (Martínez-Cañas et al., 2012), prominently suppliers, customers and competitors of both.

## Materials and Methods

In order to provide a rigorous, transparent and reproducible synthesis of the literature, this article develops the method called systematic review (Gomezelj, 2016). The most suitable documents were chosen according to a process that allows to identify existing research, to examine the value and relevance of the research topic and, finally, to collect independent works (even with opposite results) and to summarize their implications (Becheikh et al., 2006).

For the information search, we have used Web of Science (WoS) bibliographic database, which is one of the most relevant in the field of Social Sciences, in particular in business and economics. In addition, this database is commonly used in bibliometric studies in the fields of management and organization (Dzikowski, 2018).

In order to choose the publications to include in the systematic review and to exclude the subjectivity of the researchers in the data collection, we have followed a keyword search of the literature.

Articles, proceedings papers, book chapters and reviews published in peer-reviewed journals were the result of the keyword search in the WoS database. The following were the keywords: ‘industrial clusters,’ ‘industrial districts,’ and ‘global value chains.’ The search took into consideration works published from 1900 to January 2020, according to Table 2. The total number of documents was 246. After the filtering process, a total of 155 documents were selected.

**TABLE 2:** Data collection.

Database	Web of Science (WoS)	
Geographical range	Global scientific production	
Features	JCR impact factor Immediacy index Citations Quartile	
Search criteria	Topic	
Inclusion criteria	Article Proceedings paper Book chapter Review	
Time frame	All years to 2020	
Search date	15 January 2020	
Keywords	TS = (industrial cluster* OR industrial district* AND global value chain*)	
Initial documents	246	
Refinement	Business Economics Management	Duplicated documents No author Not topic related
Final documents	155	

## Results

This section describes the results found in the sample of documents analyzed on current research in industrial clusters and global value chains. Firstly, evaluative techniques are presented in Section “Bibliometric Evaluative Techniques,” including measures of productivity and impact metrics as citation analysis. Secondly, Section “Bibliometric Relational Techniques” focuses on relational techniques, as the co-citation analysis and the co-word analysis.

### Bibliometric Evaluative Techniques

The academic impact and relative influence of research on industrial clusters and global value chains is measured through the use of evaluative techniques. Accordingly, productivity is assessed based on the historical evolution of publications, the distribution of articles by journal or book and also by individual author (Ospina-Mateus et al., 2019). In addition, the most cited documents allows us to evaluate the impact or relative influence of the topic (Hall, 2011). These techniques were developed using SciMAT software.

#### Productivity Assessment

**FIGURE 1:**
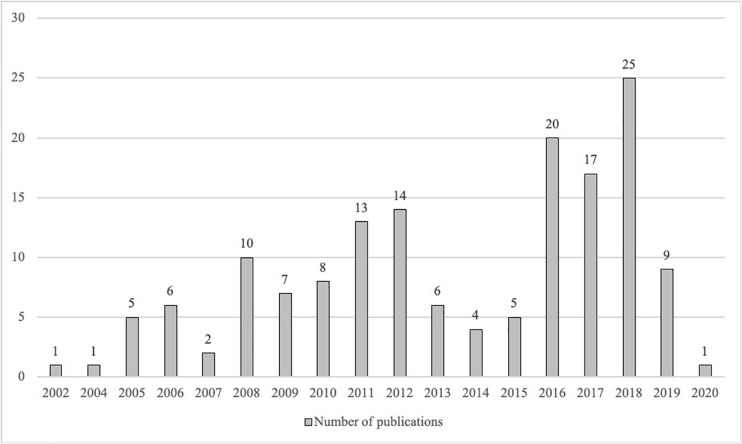
Historical evolution of publications.

Figure 1 shows the total number of documents published in the field of study for the period 1990 to January 2019.

The first publication within these topic appeared in 2002 in the Journal of Regional Studies (Humphrey and Schmitz, 2002). The highest peak of publications took place in 2018 (*n* = 25). The figure shows the presence of a growing interest in the topic, though the pace of publications is not steady, with some periods of shortage, remarkedly the years 2013–2015. Two different reasons can explain this situation. On the one hand, the impact of special issues, or books like the one edited by De Marchi et al. (2017b), is important in shaping the academic production pace, as they gather the participation of some of the most prominent authors and research teams. On the other hand, we can state that this field is relatively young, and some irregularities can be expected in this kind of topics, as Lange et al. (2011) proved for another then young topic, the study of corporate reputation.

All these 155 documents were included in 98 publications. Particularly, 81 of them (83%) published one paper; 9 (9%) published 2 papers; and 8 publications (8%) included more than 2 documents. Journals with more than three articles on the research topic are listed in Table 3.

**TABLE 3:** Distribution of articles by journal or book and impact factor.

Journal	Documents	Impact Factor (2018)/Categories
Entrepreneurship and Regional Development	12	2,928 (JCR) Development Studies (Q1) Business (Q2)
Local clusters in global value chains: linking actors and territories through manufacturing and innovation	9	Book
Regional Studies	8	3,074 (JCR) Economics (Q1) Geography (Q1) Environmental Studies (Q2) Regional and Planning (Q2)
Journal of Business Ethics	8	3,796 (JCR) Business (Q1) Ethics (Q1)
World Development	7	3,905 (JCR) Development Studies (Q1) Economics (Q1)
Journal of Economic Geography	5	3,359 (JCR) Economics (Q1) Geography (Q1)
Competition and Change	4	0,793 (SJR) Business, Management and Accounting (miscellaneous) (Q1)

Apart from the monography referred in Table 3, six journals have gathered 47 articles, which means around one third of the ones finally included in the revision. We can find some communalities between these journals, such as being high quality publications, included in the first quartile of the Journal of Citation Report (JCR), or using mostly a regional or geographical approach. Nevertheless, we can distinguish between *economics* journals, such as World Development or the Journal of Economic Geography; and *business and management* journals, like the Journal of Business Ethics or Competition and Change. This distinction will also be present in the analysis of the keywords used by the authors.

There is a total of 276 authors for the 155 selected documents. It should be highlighted that the highest share of authors (87.7%; *n* = 242/276) is related to a single publication, and 12.3% (*n* = 34/276) is attributed to two or more publications. Based on the total number of publications, Table 4 presents the most productive authors —more than two articles—. P.Lund-Thomsen is the most productive author.

**TABLE 4:** Distribution of articles by individual author.

Author	Title	Journal	JCR Impact Factor (2018)/ Categories	Year	Docs.
Lund-Thomsen and Nadvi, 2010b	Global value chains, local collective action and corporate social responsibility: a review of empirical evidence	Business Strategy and the Environment	6,381 (JCR) Business (Q1) Management (Q1) Environmental Studies (Q1)	2010	8
Lund-Thomsen and Nadvi, 2010a	Clusters, chains and compliance: Corporate social responsibility and governance in football manufacturing in South Asia	Journal of Business Ethics	3,796 (JCR) Business (Q1) Ethics (Q1)	2010	8
Lund-Thomsen and Pillay, 2012	CSR in industrial clusters: An overview of the literature	Corporate Governance: The International Journal of Business in Society	0,43 (SJR) Business, Management and Accounting (miscellaneous) (Q2)	2012	8
Lund-Thomsen et al., 2016b	Special issue on industrial clusters and corporate social responsibility in developing countries	Journal of Business Ethics	3,796 (JCR) Business (Q1) Ethics (Q1)	2016	8
Pyke and Lund-Thomsen, 2016	Social upgrading in developing country industrial clusters: A reflection on the literature	Competition and Change	0,793 (SJR) Business, Management and Accounting (miscellaneous) (Q1)	2016	8
Lund-Thomsen et al., 2016a	Industrial clusters and corporate social responsibility in developing countries: What we know, what we do not know, and what we need to know	Journal of Business Ethics	3,796 (JCR) Business (Q1) Ethics (Q1)	2016	8
Fayyaz et al., 2017	Industrial clusters and CSR in developing countries: the role of international donor funding	Journal of Business Ethics	3,796 (JCR) Business (Q1) Ethics (Q1)	2017	8
Gulati et al., 2018	Cluster matters: corporate social responsibility and micro, small and medium-sized enterprise clusters in India	Research Handbook on Small Business Social Responsibility	Book		8
Bettiol et al., 2008	Networks, technologies and globalization processes in SMEs: the Italian case	European Conference on Information Management and Evaluation	Conference	2008	7
Chiarvesio and Di Maria, 2009	Internationalization of supply networks inside and outside clusters	International Journal of Operations and Production Management	4,111 (JCR) Management (Q1)	2009	7
Bettiol et al., 2017	Industrial District Firms Do Not Smile: Structuring the Value Chain between Local and Global	Breaking up the Global Value Chain: Opportunities and Consequences	Book	2017	7
De Marchi et al., 2017c	New frontiers for competitiveness and innovation in clusters and value-chains research	Local Clusters in Global Value Chains	Books	2017	7
De Marchi et al., 2017a	Industrial districts, clusters and global value chains: toward an integrated framework.	Local Clusters in Global Value Chains	Book	2017	7
Bettiol et al., 2018b	Manufacturing, where art thou? Value chain organization and cluster-firm strategies between local and global	Local Clusters in Global Value Chains	Book	2018	7
Bettiol et al., 2018a	Does It Pay to Be International? Evidence from Industrial District Firms	Contemporary Issues in International Business	–	2018	7
Sammarra and Belussi, 2006	Evolution and relocation in fashion-led Italian districts: evidence from two case-studies	Entrepreneurship and Regional Development	2,928 (JCR) Development Studies (Q1) Business (Q2)	2006	5
Belussi, 2011	The new Marshallian districts and their process of internationalization	Handbook of regional innovation and growth	Book	2011	5
Belussi and Sedita, 2012a	18 Multiple Path Dependency and Creativity in Industrial Districts	Managing Networks of Creativity	–	2012	5
Belussi and Sedita, 2012b	Industrial districts as open learning systems: Combining emergent and deliberate knowledge structures	Regional Studies	3,074 (JCR) Economics (Q1) Geography (Q1) Environmental Studies (Q2) Regional and Urban Planning (Q2)	2012	5
Belussi et al., 2017	MNEs and clusters: The creation of place-anchored value chains	Local Clusters in Global Value Chains	Book	2017	5

The most prolific authors in this field of study are aligned with three different concerns. Lund-Thomsen is especially concerned by corporate social responsibility, analyzing in different papers the ethical impacts of offshoring activities related with industrial clusters of developing countries. Though the inclusion of industrial clusters located in developing countries in global value chains has been defended as a potential source of economic progress for local agents, the different scandals that have aroused reveal the need for both compliance policies and CSR concerns, driven by local agents, multinational companies linked with the clusters, external organizations or all of them. Lund-Thomsen has analyzed the role of these different agents in the design and development of actions and governance policies in order to address this problem through different papers, using mainly a case study analysis approach, as well as carrying out different reviews of the state of the art.

Di Maria, in turn, has analyzed the decisions made by companies included in a cluster when choosing between relying on local supply networks and national or international ones, comparing them with the options taken by non-cluster companies. So, the tension between local and global approaches is present along her work, analyzing the conditioning factors behind this decision. To do so, she used a mainly quantitative approach, originally based on one large database of Italian firms.

Finally, Belussi has considered the impact of global value chains on traditional industrial clusters. From her point of view, the globalization of the economy has an obvious impact on local industrial districts, which will be successful in their integration in global value chains if the firms included in them are able to learn and assume new activities beyond manufacturing. The approach used to analyze this situation uses mainly case studies.

#### Impact Assessment: Citation Analysis

The most prominent documents in a research area are those with the highest levels of citations. Thus, citation analysis allows us to detect the influence of certain documents on a particular topic (Merigó et al., 2016). Table 5 shows the documents with the highest number of citations in absolute terms.

**TABLE 5:** The top five most cited documents.

*R*	Title	Author/s	Journal	Impact Factor (2018)/	Year	TC	C/Y
1	How does insertion in global value chains affect upgrading in industrial clusters?	Humphrey and Schmitz, 2002	Regional Studies	3,074 (JCR) Economics (Q1) Geography (Q1) Environmental Studies (Q2)	2002	843	46,8
2	Upgrading in global value chains: lessons from Latin American clusters	Giuliani et al., 2005	World Development	3,905 (JCR) Development Studies (Q1) Economics (Q1)	2005	363	24,2
3	Clusters, connectivity and catch-up: Bollywood and Bangalore in the global economy	Lorenzen and Mudambi, 2013	Journal of Economic Geography	3,359 (JCR) Economics (Q1) Geography (Q1)	2013	140	20,0
4	Beyond strategic coupling: reassessing the firm-region nexus in global production networks	MacKinnon, 2012	Journal of Economic Geography	3,359 (JCR) Economics (Q1) Geography (Q1)	2012	157	19,6
5	The changing global geography of low-technology, labor-intensive industry: clothing, footwear, and furniture	Scott, 2006	World Development	3,905 (JCR) Development Studies (Q1) Economics (Q1)	2006	114	8,1

*R, rank; TC, total citations; C/Y, citations per year.*

The most cited paper was published in the Journal of Regional Studies 18 years ago. This work, that gave birth to this research topic, Humphrey and Schmitz (2002), is the most cited one both in absolute (843 cites) and relative (46,83 cites per year) terms.

This paper can be considered the seminal work of the main research stream inside this topic: the upgrading of local clusters through their inclusion in global value chains and environments. The relevance of this specific line, mainly related with the balance between regional development and globalization, is so clear that at least four of the five top cited articles address it. The concern about ethics and CSR is also present in most of these articles.

### Bibliometric Relational Techniques

The relational bibliometric techniques — co-citation and co-word analysis— allow the structure of the research topic to be defined according to the main themes and topics discussed (Zupic and Čater, 2015). There are other relational techniques such as co-authorship and bibliographic coupling, which are not suitable for this article considering the limited number of documents and the forward-looking approach of the study. A bibliometric mapping tool — VOS-Viewer software— is used to provide a visual overview of the analysis (Van Eck and Waltman, 2010).

#### Intellectual Structure and Thematic Organization: Co-citation Analysis

Co-citation analysis enables the identification of interrelated influential works and, consequently, the intellectual structure and thematic organization of a research topic. Two elements are co-cited when they are cited together in another article, which means that there is a thematic similarity and affinity between them. Depending on the unit of analysis, several types of citations are applied in this article: document co-citation; author co-citation; and journal or source co-citation (Small, 1973; White and McCain, 1998; Shafique, 2013).

**FIGURE 2:**
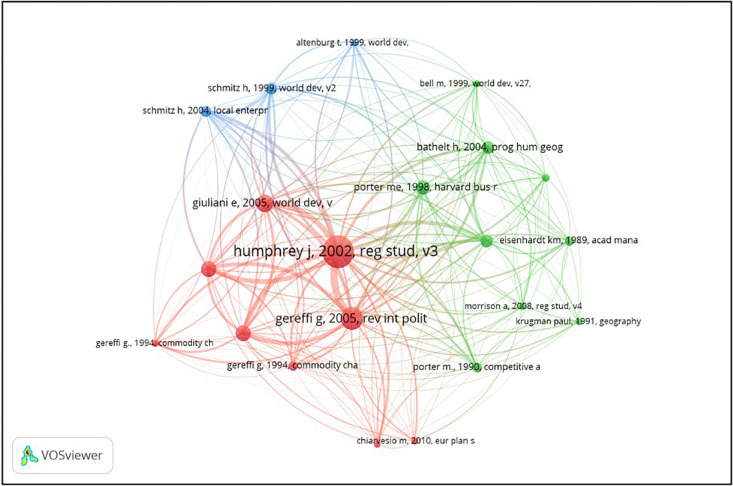
Co-citation of documents in the field of industrial clusters and global value chains.

The document co-citation allows the identification of the studies on industrial clusters and global value chains that are most frequently cited together (Small, 1973). Of the 155 documents selected, we have identified 7,226 references cited. Of this total, 21 met the minimum threshold of 15 citations per article. As can be seen in Figure 2, the largest group is composed of 9 elements. The dots or nodes illustrate the references, the larger the number of citations per document, the larger the node size. The links between the nodes represent co-citation relations. The strength of the link is illustrated by the thickness of the link. According to the association strength normalization method used by the VOS-Viewer, we have found three clusters.

Being their previous works more than relevant precedents, we can place Humphrey and Schmitz (2002) and Gereffi et al. (2005) papers in the very origin of the studies about the upgrading of clusters through their participation in global value chains. Some of the most prominent papers in this topic are co-authored by them, and the work of the teams in which they participate is clearly a must when it comes to the analysis of this business trend.

These authors reached the study of the role of local clusters in global value chains starting from different points. The previous publications of Gereffi, frequently cited in the topic, deal with the transformations experimented by commodity chains, pushed towards globalization due to the demand of the buyers (buyer-driven commodity chains). Therefore, the integration of local clusters in these dynamics was a topic to be boarded. Schmitz, in turn, developed previous research about local industrial clusters placed in developing countries, analyzing the conditions that could foster their growth. Probably, the most important one is precisely their inclusion in global supply chains. So, either starting from the study of the globalization of the supply chains or stemming from the upgrading of local clusters, the topic analyzed in this paper appears as a logical meeting point.

The green cluster includes some basic references in business management about competitiveness, such as Porter (1990), or case study methodology, mainly Eisenhardt (1989), which is frequently used as foundations of many papers not necessarily dealing with this topic.

**FIGURE 3:**
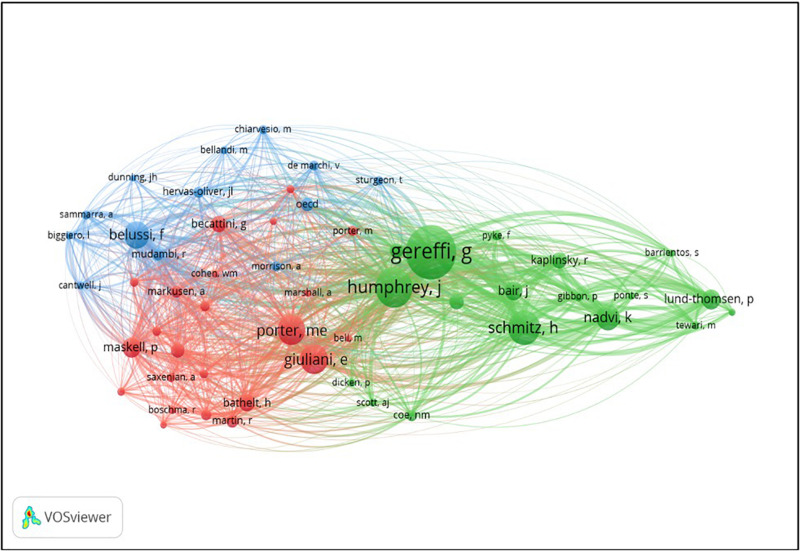
Co-citation of individual authors in the field of industrial clusters and global value chains.

Regarding the author co-citation (White and Griffith, 1981), the 155 documents selected included 4,590 cited authors. Of this total, 54 met the minimum threshold of 20 citations per author (see Figure 3).

The red cluster encompasses 23 authors, who mainly address basic literature related to the competitiveness of different types of agglomerations (industrial districts, clusters, etc.). Innovation is stressed among the relevant drivers of success studied by the authors (e.g., Giuliani, 2007). In addition, it is highlighted the role of the learning process and knowledge creation as another key source of competitive advantage in industrial clusters (e.g., Maskell and Malmberg, 1999; Maskell, 2001). It is especially relevant to stress the contribution of G. Becattini (52 citations), who after the seminal approaches to industrial districts (Marshall, 1923), rediscovered this form of organization as a socio-territorial entity (e.g., Becattini, 1990, 2004). It should also be highlighted the reference to the work of M. Porter (102 citations), who is the world’s leading authority on competitive advantage. The studies of Porter examine the competitiveness of firms and industries in global markets, and what drives the economy of an entire country (e.g., Porter, 1990, 2008).

The green cluster comprises 18 items, and it includes the authors with the highest number of citations, like Gereffi G. (198 citations), Humphrey (140 citations), and Schmitz H. (119 citations). This group is focused on the analysis of value chains potentiality in internationalized and global contexts (e.g., Gereffi and Kaplinsky, 2001). The study of Humphrey and Schmitz (2002) deserves to be mentioned because of its relevance – among the 140 citations of Humphrey, 83 come from these work. This a key research since it addresses how the integration in global value chains impacts on the enhancement of industrial clusters, which can be considered one of the cornerstones of this research topic, as stated before.

Finally, the blue cluster encompasses 13 authors and combines the topic of industrial clusters and global value chains. The most prominent author is Belussi F. (90 citations), who focuses his research on the Italian industrial clusters (e.g., Belussi, 2003; Belussi and Sedita, 2009). Mudambi addresses the tracking and tracing of diverse stages of the value chain depending on the results to be obtained (e.g., Mudambi, 2008; Mudambi and Venzin, 2010). Finally, both Belussi F. and Hervas-Oliver J.L. (33 citations) pay special attention to the generation and diffusion of knowledge (e.g., Belussi, 2003) and the particular dynamics of multinational enterprises (MNEs) (e.g., Hervas-Oliver and Albors-Garrigos, 2008; Hervas-Oliver and Boix-Domenech, 2013).

**FIGURE 4:**
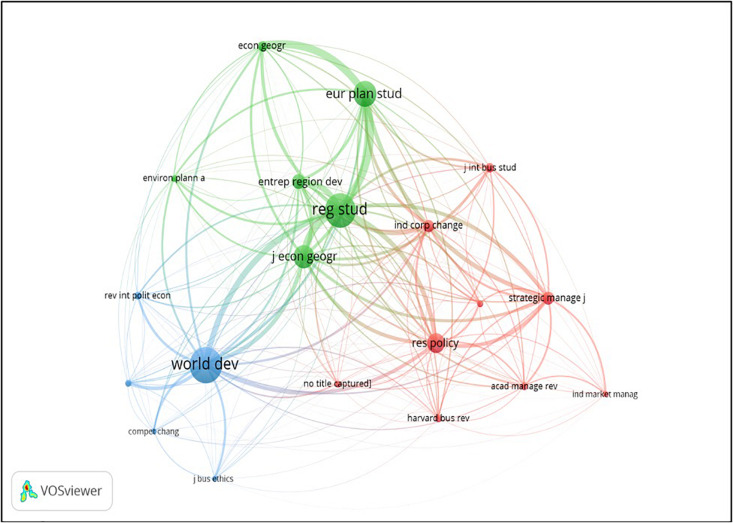
Co-citation of sources or journals in the field of industrial clusters and global value chains.

The journal co-citation allows the identification of the journals publishing on industrial clusters and global value chains that are most frequently cited together (McCain, 1990). The journal research areas are similar when they are frequently cited together. Of the 155 documents selected, we have identified 3,621 journals cited. Of this total, 20 met the minimum threshold of 63 citations per source. As shown in Figure 4, the gap between two journals represents their relatedness in terms of co-citation linkages. The most relevant co-citation links are also illustrated with thicker lines.

The previous analysis of the most prolific journals showed that, though the concern about regional development was always present, two different perspectives (the economic and the management ones) could be addressed. This relational analysis can help us to shed a closer light over this issue. According to the similarity method used by VOS-Viewer, we have identified three clusters.

The red cluster comprises journals related to the areas of Business, management, organization and strategy (e.g., Research Policy, Strategic Management Journal, etc.). So, the topics of these publications are mainly related with the opportunities for additional value creation that global value chains generate for the firms included in industrial districts. This cluster is, so, closely linked with the study of value cocreation.

The green cluster basically focuses on relevant research in economic geography as well as local and regional development (Regional Studies, Journal of Economic Geography). The main concerns of these publications are linked with the economic impact of a new industrial configuration, rebalancing the value creation between local and global tensions.

Finally, the blue cluster consists of a more varied set of journals (Journal of Business Ethics, World Development, etc…) that focus their research on economic policies and business organization, and their impact on the wellness of communities and individuals. The main considerations about the actions that both firms and governments should implement in order to protect workers and other stakeholders are addressed by these journals.

#### Conceptual Structure: Co-word Analysis

**FIGURE 5:**
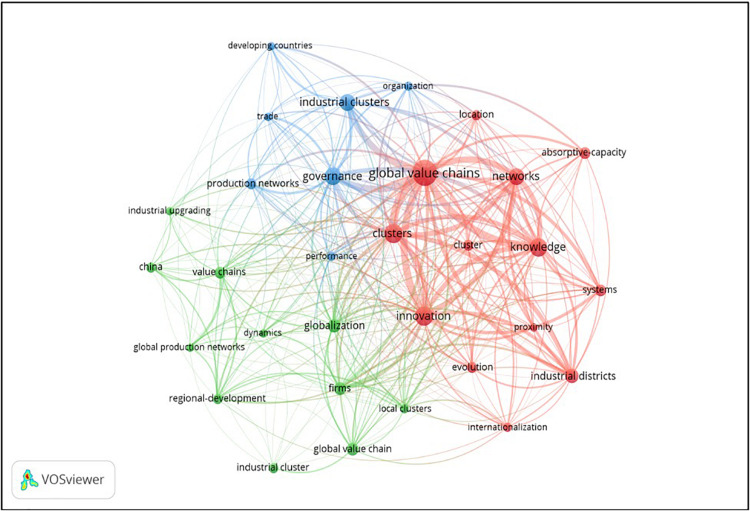
Co-occurrence network of keywords in the field of industrial clusters and global value chains.

Research trends and hot topics emerge from the co-word analysis of the most frequent keywords (Li et al., 2016). As can be seen in Figure 5, in our sample of 155 documents, we have detected 682 keywords. We have only considered the keywords that appear in at least 8 publications. The nodes illustrate the occurrence of the keywords, the greater the weight of an element, the larger the node size. The links between the nodes represent the number of times that the keywords occur together. The strength of the link is illustrated by the thickness of the link.

The main keywords in the research topic are “global value chains” (70 occurrences and 247 total link strength), “innovation” (39 occurrences and 177 total link strength) and “clusters” (41 occurrences and 170 total link strength). So, it could be said that industrial clusters and global value chain seem to be to be a hot topic, with different research lines being developed from these core concepts.

The node, “global value chains,” has stronger links with “industrial clusters,” “clusters” and “networks.” This node ties in closely with “innovation,” “governance” and “knowledge” which is obvious considering that all of them determinants of competitive advantage, and so linked with the business management perspective which was remarked when analyzing the main journals. The node “innovation,” also belonging to this red cluster of keywords, is closely related to “global value chains,” “industrial districts,” “industrial clusters,” “clusters”; “networks” and “knowledge,” remarking the innovative spillover effect of clusters and global value chains.

The map also shows the other two concerns stated before. The blue cluster can be associated with the institutional and organizational regulation of the new shapes of value systems. Therefore, words like “governance,” “industrial clusters,” or “organization” are prominent and deeply related to each other. Finally, the green cluster of keywords can be linked with the geopolitical analysis, being “globalization,” “global value chain,” and “firms” the most frequent ones, showing links with “regional development” or “global production networks.” The relatively lower presence of these keywords could point at a relatively higher potential for further development of these kind of studies.

As can be seen, industrial clusters and global value chains remain an emerging research field that requires additional investigation and discussion. An interesting development of this study will probably be to separate bibliometric analysis into 5-year stages. Considering the limited number of publications and their concentration in recent years, this is not possible at present.

## Discussion and Conclusion

The traditional configuration of a value system composed by independent companies working isolatedly is less frequent every day. From a service-dominant logic, value in not created along different independent steps, but through the co-production and the value in use of different agents involved in a service system (Ranjan and Read, 2016).

Industrial clusters have been studied as a productive combination of competition and cooperation amongst companies related with a specific activity (Giuliani et al., 2005), creating an adequate environment for value cocreation (Crick et al., 2020). Nevertheless, clusters had always been mainly rooted to the ground, anchored to a specific location. Different studies, amongst which Humphrey and Schmitz (2002) must be highlighted, proposed that the inclusion of these industrial districts into global value chains (Gereffi, 1999; Giuliani et al., 2005) could result into their upgrade, generating additional opportunities of value creation.

This article aims to provide a summary of research on industry clusters and global value chains through a systematic review taking into account the previously published literature. Through a bibliometric analysis, this paper disentangles the intellectual and conceptual structure of the joint research of the topics of industrial clusters and global value chains.

The results show that we are facing an interesting topic that has been able to capture a high degree of attention of the academic community in less than 20 years. Starting from the seminal work of Humphrey and Schmitz (2002), still the most cited one in this research field, the evolution of the number of publications along this time reveals a clearly growing interest, despite of the classical sawtooth of relatively young research streams.

The original research question, which is the main concern of some of the most cited articles, deals with how to upgrade the potential of industrial clusters integrating them into global value chains (Humphrey and Schmitz, 2002; Giuliani et al., 2005). This original concern immediately leads the studies in two complementary directions: its effect over the firm competitiveness, especially related with business management; and its impact on regional development, potentially modifying the balance between local and global tendencies. Additionally, a third main research line has aroused, specially fueled by the works of the most prolific author in the field, Lund-Thomsen, dealing with the ethical aspects that should be addressed by local agents when cooperating with partners established abroad, that have to be approached by institutional policies and corporate governance.

The presence of these three main intertwined research lines can be seen along the results drawn by all the techniques used in our bibliometric analysis. The most productive journals are related with these areas, especially with regional development; the authors and articles cited can be closely linked with their theoretical and empirical foundations; and the keywords used by the different authors are some of the most familiar terms in these literatures.

Probably, the main limitation of the paper derives from the necessary selection of documents to be analyzed. Though the database chosen, WoS, gathers the majority of the most prominent publications in this research stream, some relevant studies could be potentially not included in it. Also, even if it is not frequent, some of the documents analyzed may not include keywords, which could slightly modify the results of the co-work analysis. Finally, due to the nature of this kind of analyses, the interpretation of the different maps is unavoidably subjective.

The relative youth of the topic has also put some limits to the analyses carried out. For instance, it is still not possible to distinguish different periods in the research of this field, as long as the majority of the scientific production belongs to the latest years. So, if the literary production continues to increase, and the production measurements clearly point at that possibility, it will be interesting to carry out a new study in some years in order to analyze the possible evolution of the stream of research. This is clearly one of the future research lines concerning clusters and global value chains, as well as a deep literary review that can shed an additional light on this literature.

Behind the entire topic, no matter the specific focus we consider, we can find the tension between local and global tendencies. The integration of clusters in global value chains has created an opportunity to balance both opposite trends, generating different issues to be analyzed and solved, present in different ways in the papers included in this analysis. Nevertheless, we can never say that this upgrading is the ultimate, or even the best, synthesis of both.

The burst of the crises of COVID-19 has put again this debate on the table. Are the countries generating an excessive external dependence when it comes to key industries? Or is, in turn, the solution to the crisis closer to higher levels of international cooperation and integration? The answers given to these questions will, in turn, decisively influence the decisions and behavior of the economic agents, leading to new breakeven points which can be closer to one or the other, local or global, end.

## Data Availability Statement

The raw data supporting the conclusions of this article will be made available by the authors, without undue reservation.

## Author Contributions

TG-T: conceptualization, methodology, and supervision. TG-T and J-LR-S: writing—original draft preparation. AM-N and RG-L: writing—review and editing. RG-L and J-LR-S: visualization. AM-N: project administration. J-LR-S, TG-T, AM-N, and RG-L: validation. TG-T and AM-N: formal analysis. RG-L: resources. All authors contributed to the article and approved the submitted version.

## Conflict of Interest

The authors declare that the research was conducted in the absence of any commercial or financial relationships that could be construed as a potential conflict of interest.
